# Relationship of Salivary Lactoferrin and Lysozyme Concentrations with Early Childhood Caries

**DOI:** 10.15171/joddd.2015.022

**Published:** 2015-06-10

**Authors:** Masoumeh Moslemi, Mandana Sattari, Fahimeh Kooshki, Faezeh Fotuhi, Neda Modarresi, Zahra Khalili Sadrabad, Mohammad Saeid Shadkar

**Affiliations:** ^1^Professor, Department of Pediatric Dentistry, Faculty of Dentistry, Shahid Beheshti University of Medical Sciences, Tehran, Iran; ^2^Professor, Department of Immunology, Shahid Beheshti University of Medical Sciences, Tehran, Iran; ^3^Assistant Professor, Department of Pediatric Dentistry, Faculty of Dentistry, Shahid Beheshti University of Medical Sciences, Tehran, Iran; ^4^Assistant Professor, Department of Pediatric Dentistry, Faculty of Dentistry, Yazd University of Medical Sciences, Yazd, Iran; ^5^Pedodontist, Private Practice, Tehran, Iran; ^6^Post-graduate Student, Department Pediatric Dentistry, Faculty of Dentistry, Tabriz University of Medical Sciences, Tabriz, Iran; ^7^PhD Student in Economics, Faculty of Economics, Tehran University, Tehran, Iran

**Keywords:** Dental caries, lactoferrin, lysozyme, saliva

## Abstract

***Background and aims.*** Lysozyme and lactoferrin are salivary proteins which play an important role in innate defense mechanisms against bacteria. This study investigated the association of salivary lysozyme and lactoferrin concentrations with early childhood caries (ECC).

***Materials and methods.*** This study was carried out on 42 healthy children (age range, 36 to 71 months), of whom 21 were caries free (CF) and 21 had ECC. Disposable needle-less syringes were used to collect unstimulated saliva from buccal and labial vestibules. Fifteen children who had ECC were treated completely and their saliva was collected in the same way for the second time, three months after treatment. Lysozyme and lactoferrin concentrations were measured and recorded by the ELISA method. The intergroup comparisons were carried out using chi-square, Student’s t-test and Wilcoxon signed ranked test. A P-value less than 0.05 was considered as statistically significant.

***Results.*** The mean concentration of lysozyme was significantly higher in CF group compared with that of ECC group (P = 0.04). Although the mean concentration of lactoferrin in ECC group was higher in comparison with ECC group, the difference was not statistically significant (P = 0.06). After dental treatment, the mean concentrations of lysozyme and lactoferrin did not change in comparison with their concentrations before treatment.

***Conclusion.*** ECC may have a relationship with lower concentrations of unstimulated salivary lactoferrin and lysozyme and reduced amounts of these two salivary proteins may be a risk factor for dental caries in children.

## Introduction

Dental caries is a common chronic infectious transmissible disease resulting from tooth-adherent specific bacteria, primarily *Streptococcus mutans*, that metabolize sugars to produce acid which, over time, demineralizes tooth structure.^1^ According to AAPD definition, the disease of early childhood caries (ECC) is the presence of one or more decayed, missing or filled tooth surfaces in any primary tooth of a seventy-one month-old child or younger.^2^ It is recognized as a dull, white demineralized enamel that quickly advances to obvious decay along the gingiva.^3^ The caries attack usually starts on the labial surface of the upper anterior incisors.^4^

The consequences of ECC can affect the immediate and long-term quality of life for the child and the family, and can have significant social and economic consequences beyond the immediate family as well.^5^ Untreated caries are associated with pain and can lead to problems with speech, sleeping, and eating in children.^6^ Moreover, children with severe caries may experience reduced weight and delayed growth.^7^

ECC is a multifactorial disease. Breastfeeding and many other biological variables, such as *S. mutans*, enamel hypoplasia, intake of sugars, as well as social variables, such as parental education and socioeconomic status, may have important roles in initiation and progression of ECC.^5^ Current research is focused on the development of preventive strategies against caries and the identification of risk factors as well as natural oral defenses.^8^ Saliva has an essential role in caries prevention, through functions relying on physicochemical characteristics such as flow rate, pH and buffering capacity, and therefore, variations under threshold levels are considered as risk factors for the development of dental caries.^9^ Saliva contains many innate or acquired (antibodies) defense mechanisms. Antimicrobial proteins including lysozyme, lactoperoxidase system, lactoferrin, and high weight molecular glycoproteins are among the innate or unspecific immunological factors.^10,11^

Lysozyme is an antibacterial enzyme which is in rather high concentrations in body fluids such as serum/plasma, amniotic fluid, saliva, and tear, and also in lesser concentrations in urine, bile, and CSF. This enzyme has been found in phage cell granules and it is assumed to be a powerful antibacterial agent against G+ bacteria.^12^ In the oral cavity, lysozyme is secreted from major and minor salivary glands, gingival crevicular fluid, and salivary leukocytes. Salivary lysozyme has muramidase activity, i.e. is capable of hydrolyzing the B (1->4) bond between N-acetylmuramic acid and N-acetylglucosamine in the peptidoglycan layer of the bacterial cell wall, and thus, hydrolyzes the polysaccharide wall of bacteria in various situations such as hypoosmolarization.As a strongly cationic protein, lysozyme has also been shown to mediate bacterial aggregation and adherence and to activate bacterial autolysins which destroy the bacterial cell walls.^13,14^

Lactoferrin is a non-enzymatic antibacterial protein. It is widely spread in body fluids such as saliva and tear, and even in secondary granules of polymorphonuclear leukocytes. Bee sting contains the highest concentration of lactoferrin. It is secreted by the serous cells of the major and minor salivary glands. It has an iron-chelating property which deprives microorganisms of this essential element. Lactoferrin in its iron-free state is known as apo-lactoferrin, and is capable of antibacterial activity via the direct binding of bacteria to lactoferrin and agglutinating *S. mutans* thus allowing ease of removal of the agglutinated bacteria from the oral cavity via mechanical action of saliva and the swallowing of the agglutinated bacteria. In addition, lactoferrin has demonstrated potent antiviral, antifungal and antiparasitic activity, towards a broad spectrum of species.^15^ Lactoferrin exhibits *in vitro* anti-inflammatory activities and several domains are present within its polypeptide chain that demonstrate antimicrobial effects.^1^

Lactoferrin can also bind to salivary agglutinin, suggesting that both salivary proteins can act together to bind microbes.^13^ Because of its antibacterial activities, lactoferrin is used as a component in mouthwashes and dental creams.

Numerous studies in the past have attempted to relate certain aspects of salivary output and composition to caries susceptibility. Mass et al^16^ found an association between low lysozyme levels and decreased numbers of *S. mutans* and lactobacilli.^16^ Felizardo et al^17^ reported a slight association between lysozyme concentrations and DMFT; however, lactoferrin was positively correlated with both DMFT and restored teeth.^17^ It has been reported that the decayed surface index rose with increases in the levels of lactoferrin in 15-years old children,^18^ and Stuchell & Mandel^19^ found that there was essentially no difference between the caries-free and caries-active groups in lysozyme concentrations. In a two year cohort study, it was found that none of the single salivary antimicrobial agents such as lysozyme or lactoferrin has sufficiently strong power to have diagnostic significance in vivo with respect to future caries.^20^ On the other hand, a recent study showed the high efficacy of a toothpaste containing lactoferrin, lysozyme, and lactoperoxidase in reducing the salivary levels of *S. mutans* and *L. acidophilus* in children with S-ECC.^21^ In another study, *S. mutans* and *L. casei* were inhibited by lysozyme while not affected by lactoferrin and by the synergic use of both proteins.^10^

The available evidence demonstrates conflicting and inconclusive results; some studies have reported the resemblance of salivary protein profiles between caries-active samples and caries-free controls, while the others have shown significant difference between the two groups. Moreover, very few studies concerning the association of these salivary proteins with ECC exist and more studies are needed. It is proposed that the oral microflora present an antigenic challenge, in response to which antimicrobial salivary proteins like lysozyme and lactoferrin are released.^13,22^ Hence, it was hypothesized that salivary levels of these two proteins would change once the caries were successfully treated and the ecologic balance was restored in children with ECC. Therefore, this study investigates the relationship between salivary lactoferrin and lysozyme concentrations and ECC, as well as the effect of the comprehensive dental treatment on concentrations of lactoferrin and lysozyme in saliva.

## Materials and Methods

### Subjects 

A total of 42 healthy children (21 caries-free and 21 with ECC), whose age ranged between 36 and 71 months, were included in this cross-sectional, longitudinal study. These children were selected with a convenience sampling procedure from Shahid Beheshti kindergarten and the children who were referred to the Department of Pediatric Dentistry at Shahid Beheshti University of Medical Sciences, Tehran, Iran, from 2009 to 2010. The sample size was determined using Minitab 14 computer software with consideration of α= 0.05 and β= 0.2. Children with the history of prior medication over the previous month, tooth extraction, or presence of any systemic illness were excluded.

### Clinical Examinations

Clinical examination of all subjects was carried out by a single examiner. Children with one or more decayed surface in deciduous teeth (cavitated or non-cavitated) were put in ECC group, and those absolutely free from caries were recognized as caries-free (CF) group. Examinations were carried out using artificial light, flat mirror, and explorer and were performed according to the World Health Organization diagnostic criteria, 1986.^23^ Before the commencement of the study, the parents received detailed information about the study and informed consents were obtained. They were asked to perform regular oral hygiene procedures after breakfast and prevent their children from eating and drinking one hour prior to saliva collection.

### Saliva Collection

Unstimulated saliva of all subjects was collected in a quiet room in the morning between 9 and 11 a.m. with the use of disposable needle-less syringes from buccal and labial vestibules.^24^ One to two milliliter of collected saliva of each subject was poured into coded capped microtubes which were stored in ice box to prevent hydrolysis of salivary proteins, and were sent immediately to the immunology laboratory. From 21 children with ECC, two cases because of migration and four cases because of failing to complete follow-up period were excluded. The other fifteen children received complete treatment and unstimulated saliva from this group was collected again, 3 months after the first collection.

### Laboratory Procedures

The samples were stored at −20°C. In the laboratory, all the samples were placed in room temperature, to be liquefied. After that, microtubes were centrifuged with centrifugal casting machine (Ependorf centrifuge, 5415, Germany), 10000 race/min, pending 15 min. During this process, all the probable particles over the liquid of microtubes were gently omitted and transmitted to other microtubes, and then, all procedures were carried out according to the lysozyme kit protocol (Human lysozyme, ELISA demeditec diagnostic, Germany) and the lactoferrin kit protocol (human lactoferrin ELISA, Bethyl laboratories , USA). The optical density of samples was read at 450 nm and the lysozyme and lactoferrin concentrations (ng/ml) were determined according to the manufacture’s instruction.

### Statistical Analysis

The intergroup comparisons were carried out using chi-square, Student’s t-test and Wilcoxon signed ranked test. The SPSS 11.0 software package was used and a P-value less than 0.05 was considered as statistically significant.

## Results

The initial 42 samples, consisting of 22 boys and 20 girls, showed no gender difference between the two study groups (P < 0.05, chi-square test).

Mean concentration of salivary lysozyme in ECC group was 2180 ng/ml whereas mean lysozyme concentration was 9573.81 ng/ml in CF group. The Student’s t-test demonstrated a P-value of 0.04 on comparing the groups, and therefore, the difference between the salivary lysozyme amounts of two groups was found to be statistically significant (P < 0.05; Table 1). Mean concentrations of salivary lactoferrin in ECC and CF groups were 37.9 ng/ml and 50.93 ng/ml, respectively. The difference between the two groups was not statistically significant (P = 0.06, Student’s t-test; Table 2). After treatment, the mean concentrations of salivary lysozyme and lactoferrin among the treated children were 2108 ng/ml and 42 ng/ml respectively, which were not significantly different compared to that of ECC group prior to intervention (P=0.86 and P=0.2, respectively, Wilcoxon signed rank test; Tables 3 & 4).

**Table 1:** Salivary concentration of lysozyme (mean ± SD) in ECC and CF groups

**Study groups**	**Number of children**	**Lysozyme concentration**	**P value**
**ECC**	21	2180 ± 653.52	0.04
**CF**	21	9573.81 ± 1148.3	
ECC, early childhood caries; CF, caries free.			

**Table 2:** Salivary concentration of lactoferrin (mean± SD) in ECC and CF groups

**Study groups**	**Number of children**	**Lysozyme concentration**	**P value**
**ECC**	21	37.9 ± 16.43	0.06
**CF**	21	50.93 ± 24.44	
ECC, early childhood caries; CF, caries free.			

**Table 3:** Salivary concentration of lysozyme in ECC group, before and after treatment

**Study groups**	**Number of children**	**Lysozyme concentration**	**P value**
**Treated ECC**	15	2108 ± 59.1	0.86
**ECC before treatment**	21	2180 ± 653.52	
ECC, early childhood caries.			

**Table 4:** Salivary concentration of lactoferrin in ECC group, before and after treatment

**Study groups**	**Number of children**	**Lysozyme concentration**	**P value**
**Treated ECC**	15	42 ± 11.62	0.2
**ECC before treatment**	21	37.9 ± 16.43	
ECC, early childhood caries.			

## Discussion

In the present study, it was demonstrated that the salivary lysozyme level was significantly higher in CF group compared to that of ECC group. Lactoferrin concentration was also higher in CF group in comparison with ECC group; however, the difference was not statistically significant. After treatment, the amounts of salivary lactoferrin and lysozyme did not change compared to their concentrations before treatment.

Dental caries remains a widely prevalent bacterial infection especially among developing countries. There are several risk factors considered for dental caries; some are related to saliva and bacterial colonization on the dental biofilm. Saliva is a complex body fluid that provides a general protective mechanism for the oral cavity tissues. Flow rate, buffer capacity, inorganic components, and antimicrobial factors such as lysozyme and lactoferrin are among the special characteristics of saliva, which may have important roles in dental caries prevention. Saliva-based prediction of caries risk may lead to development of new preventive strategies against caries and more importantly, against ECC. Numerous studies have investigated the correlation between salivary proteins and caries experience; however, there are conflicting views regarding the relationship between salivary proteins and dental caries. The treatment of dental caries and its correlation with the change in salivary lactoferrin and lysozyme levels has not been well-established.

In this study, ELISA method was used in order to measure lactoferrin and lysozyme concentrations with high accuracy and in a short time. Because of the low number of children fulfilling the study’s inclusion criteria, we selected the samples from two separate sources which may affect our results. We found the higher lysozyme concentration in the caries-free group in comparison with ECC group and the difference was statistically significant (P < 0.05). Hao et al^25^ stated that lysozyme amount difference between two groups of caries free children and children who had dental caries (dmft>5) was not statistically significant. Using turbidimetric technique, Bai et al^26^ found that lysozyme concentration of unstimulated saliva in ECC group was significantly higher than that of caries-free group. On the other hand, Bahhla et al^8^ demonstrated that DMFT index decreased as the lysozyme concentration increased which indicates that lysozyme provides protective and antimicrobial effect, and suggests that high concentration of lysozyme inside the oral cavity should have an important role in dental caries prevention. In our study, lactoferrin concentration was higher in the caries-free group in comparison with the ECC group; however, the difference was not statistically significant. Unlike our results, the amount of lactoferrin was higher in high dmft group than that of caries-free children in the study of Hao & Lin.^25^

The higher concentrations of both lysozyme and lactoferrin in caries-active group compared to that of caries-free group which have been previously reported in some of similar studies may be due to a compensatory mechanism. It seems that in specific situations, in the presence of caries or high levels of *S. mutans*, the secretion of these two proteins, which represent a defensive mechanism, may be stimulated. The exact conditions and circumstances, under which this compensatory mechanism may act remain to be understood. Moreover, caries is a multifactorial disease and salivary risk factors represent only a fraction of all contributing factors. Salivary proteins show inter-subject variations and antimicrobial agents are dependent on salivary flow rate in an individual manner, which in turn is regulated almost exclusively by the autonomic nervous system.^27^ In addition, depending on the simultaneous extent of gingival inflammation, the crevicular exudate, provides antimicrobial agents such as lysozyme, lactoferrin, myeloperoxidase, and immunoglobulins into the whole saliva, irrespective of the caries status.^28^ Inconsistency in study design, saliva collection methods, and salivary analysis methods is also present between various studies. Furthermore, most clinical studies investigate only one protein at a time. Salivary antimicrobial proteins interact in many ways with each other. The interactions result in additive, synergistic, or inhibitory effects. Low concentrations of an individual protein may be compensated for by other proteins with similar functions. Specific sIgA has been shown to block or enhance the independent bactericidal actions of lactoferrin,^29^ however, it may also enhance bacteriostatic iron-sequestration by lactoferrin.^30^ Polycationic anti-membrane effects of lysozyme may be enhanced by peroxidase-generated hypothiocyanite,^31^ but peroxidase itself may non-specifically block bactericidal effects of lactoferrin.^29^ Because of the presence of these highly interactive proteins, it is suggested that future attempts consider multiple salivary proteins as elements of a common system and evaluate their concentrations simultaneously. Further studies are required to clarify the detailed interactions of salivary proteins and to describe how salivary proteins vary among study populations.

## Conclusion

According to the results, dental treatment does not have any noticeable effect on lysozyme and lactoferrine concentrations, and this may indicate that the amounts of these two proteins in saliva are not dependent on the presence of caries in the oral cavity. Also, ECC may have a relationship with lower concentrations of unstimulated salivary lactoferrin and lysozyme, which may act as a risk factor for dental caries in children.

## Acknowledgments

This paper is based on a M.D. Thesis which was successfully completed by Dr. Modarresi under the supervision of Prof. Moslemi and Dr. Sattari with the cooperation of the Department of Immunology, and the Faculty of Dentistry, both at Shahid Beheshti University of Medical Sciences, Tehran, Iran.
